# Quantification of Calcium Ions From the Irrigants Activated With Erbium-Doped Yttrium Aluminum Garnet (Er:YAG) Laser in the Root Dentin: An In Vitro Atomic Absorption Spectrophotometer Study

**DOI:** 10.7759/cureus.48552

**Published:** 2023-11-09

**Authors:** Dhanalakshmi P, Kiran Kumar N, K Rashmi, Biji Brigit, Shwetha R S, Sourabh T J

**Affiliations:** 1 Conservative Dentistry and Endodontics, Government Dental College and Research Institute Bangalore, Bengaluru, IND

**Keywords:** er:yag laser, removal of smear layer, chitosan nanoparticles, calcium ion removal, atomic absorption spectrophotometer with flame

## Abstract

Objective: The present study aims to assess the quantity of calcium cation eliminated from the root canal by 0.2% chitosan nanoparticles (CNPs) (Sigma Aldrich, Mumbai, Maharashtra, India) and 17% ethylenediaminetetraacetic acid (EDTA) (Pyrax 17% EDTA Solution, Pyrax Polymars, Roorkee, Haridwar, Uttarakhand, India) which are activated with an erbium-doped yttrium aluminum garnet (Er:YAG) laser (LiteTouch™, Light Instruments Ltd., Yokneam Elite, Israel) for smear layer removal using a flame atomic absorption spectrophotometer (AASF) (Deeksha Analytical Pvt Ltd, Gokula Extension, Bengaluru, Karnataka, India).

Methodology: Using the crown-down technique, 60 non-carious single-rooted premolars were instrumented with rotary files and irrigated with 3% sodium hypochlorite. Based on the type of irrigation activation used, all the specimens were arbitrarily divided into five groups with 12 teeth in each group, as follows: G1, 17% EDTA activated with Er:YAG laser; G2, 17% EDTA without laser activation; G3, 0.2% CNP activated with Er:YAG laser; G4, 0.2% CNP without laser activation; and G5, controlled-deionized water. The AASF analysis for the removal of calcium ions in the irrigants was evaluated by collecting the overall quantity of each irrigating solution from the root canals. A one-way analysis of variance (ANOVA) and a Tukey post hoc test were done to determine the AASF data.

Results: 17% EDTA activated with Er:YAG laser (130.18 ± 10.3) and 0.2% CNP activated with Er:YAG laser (121.13 ± 3.9) showed the greatest concentration of calcium ions with no statistically significant difference. The lowest concentration of calcium ions was observed in 0.2% CNPs without laser activation (118.64 ± 2.9), while 17% EDTA (125.50 ± 3.0) without laser activation showed an intermediate outcome. The control group did not remove any calcium ions.

Conclusion: The findings in the present study suggest that EDTA and CNPs, which were activated with lasers, yielded the greatest release of calcium ions equally. Hence, laser-activated CNPs can be employed for essential smear layer removal.

## Introduction

The chemomechanical preparation contributes to an outstanding endodontic procedure [1]. Failure to completely debride the canal still occurs mainly in the smear layer (SL), which harbors microorganisms within the canal of the root [2]. The limited sterile environment could be due to the root canal, which comprises complex anatomy [3]. The most popularly used chelating agent for the removal of both organic and inorganic contents of SL is ethylenediaminetetraacetic acid (EDTA). However, it causes dentinal erosion and harm to the apical region around the periodontium. Thus, the research continues to explore a more biocompatible irrigant other than EDTA [4].

Of late, chitosan has been widely used in the medical field. Silva et al.'s research on chitosan showed that it can be used as a chelation agent to dissolve the inorganic parts of SL. Because of this, it could be used as an irrigant during the final irrigation [5]. Chitin undergoes alkaline deacetylation to form chitosan, which is a natural biopolymer. It is also a nontoxic cationic with antifungal and antibacterial activity [6]. Chelators are stable complexes that are formed when metal ions adhere to ligands that have many pairs of free electrons. These chelators cause changes in the concentration of calcium and phosphorus ions within the canal. Chelating agents have a decalcifying effect on SL and root canal dentin, which is good because it exposes collagen and makes the hardest part of the root canal less hard. This helps in better instrumentation with improved SL removal due to the deeper penetration of irrigants with adequate disinfection [7].

There are two hypotheses that explain the chelating property of chitosan [8]. The bridge theory explains that several groups of amino acids present in chitosan couple with identical metal ions. The pendant theory states that binding is mainly done by only one amino group and the union of one amino group with a metal ion appears like a suspended pendant. The chelating mechanism of calcium ions present in dentin is due to one of the two mechanisms causing a reduction in the inorganic content of the SL [8]. Numerous methods for SL removal comprise sonic, ultrasonic, chemomechanical, and laser activation [9]. The delivery method and the type of irrigant will influence the efficiency of any irrigating solution to eliminate the SL [6]. Recently, for SL removal, lasers have been suggested. Previous research has shown that irrigation activation by the erbium-doped yttrium aluminum garnet (Er:YAG) laser has cleared SL from root dentin and clean dentinal tubules were observed [10]. SL removal by laser-driven irrigation is achieved through cavitation and bubble explosion [11].

Most of the research in relation to chitosan nanoparticles (CNPs) has concentrated on their antimicrobial quality, with inadequate documentation on SL removal. Even though there have been a lot of studies on how different methods and mediums get rid of SL, there haven't been many studies on how to measure the amount of calcium ions in root dentin using irrigants activated by an Er:YAG laser. So, the goal of this study was to measure how well the SL was removed by comparing the calcium ion concentration in root canals treated with or without Er:YAG (LiteTouch™, Light Instruments Ltd., Yokneam Elite, Israel) laser-activated 0.2% CNPs (Sigma Aldrich, Mumbai, Maharashtra, India) and 17% EDTA (Pyrax 17% EDTA Solution, Pyrax Polymars, Roorkee, Haridwar, Uttarakhand, India) to the calcium ion concentration in the control group.

## Materials and methods

In the current study, 60 non-carious single-rooted teeth were used. Only intact teeth extracted because of orthodontic treatment or periodontal disease with fully formed roots and patent canals were included. Teeth having roots with curvature, resorption of the root, and calcified canals were excluded. After the access opening #20 K file was used to negotiate the canal, it appeared beyond the apex, and 0.5 mm short of this measurement was considered the working length. A ProTaper rotary file (Dentsply Sirona, Charlotte, North Carolina, United States) was instrumented in the crown-down technique up to size F2 for the coronal preparation. During recapitulation, all the samples were irrigated with 3% sodium hypochlorite. Any loose dentinal chips remaining were removed with 10 ml of deionized water. Absorbent paper points corresponding to size F2 of the ProTaper rotary file were used to dry the canals. Specimens were irrigated with final irrigants for SL removal. The irrigant was delivered by means of a side-vented needle -30 gauge (Canal Clean, Biodent Co. Ltd, Paju, Gyeonggi-do, South Korea) inserted passively 2 mm short of working length with back and forth motion of 2-3 mm. To simulate the clinical condition, the apex of each tooth was closed with utility wax based on the previous study by Thapak et al. [3]. Fifteen milliliters of a falcon tube with its lid punctured was used to collect the final irrigant. Each tooth was placed with its root apex inside and crown outside in the falcon tube.

The distribution of teeth was based on the final activation they had received; there were five groups in total. Group 1 (n = 12): 17% EDTA with Er:YAG laser activation was done; group 2 (n = 12): 17% EDTA without laser activation was done; group 3 (n = 12): 0.2% CNP with Er:YAG laser activation was done; group 4 (n = 12): 0.2% CNP without laser activation was done; and group 5 (n = 12): control-final irrigation with deionized water and without laser activation was done.

An Er:YAG laser (LiteTouch™) with a wavelength of 2,940 nm was used in this study to activate the chelating agents in the canals. Chelating solutions were delivered for three minutes, passing through the entire root canal system. Continuous irrigation with 5 mL of respective chelating agents was applied manually with a conventional syringe, and the irrigating solutions in each canal were activated with an Er:YAG laser. The laser tip with a diameter of 0.4 mm (400 μm) was placed stationary in the pulp chamber while the irrigant was activated. The operating parameters used were as follows: 0.3 watts, a pulse energy of 20 mJ per pulse, and a frequency of 15 Hz. The irrigant was activated for a duration of 20 seconds with a resting period of 40 seconds, and this was repeated for three cycles. The water spray on the handpiece was turned off. The protocol followed was according to a previous study by de Groot [12].

During the resting period, the utility wax was removed from the apex of the tooth to collect the irrigants that passed through the complete root canal system, passing out through the patent foramen into a falcon tube, which was subjected to an atomic absorption spectrometric study. This total volume of the collected solutions was used for the quantification of calcium ions. Chitosan powder weighing 0.2 g was mixed with 1% acetic acid in a 100 ml volume using a magnetic stirrer for two hours to receive a uniform solution.

Atomic absorption spectrophotometer (AASF) (Deeksha Analytical Pvt Ltd, Gokula Extension, Bengaluru, Karnataka, India) is one of the most common techniques for detecting metals in environmental samples. It consists of hollow calcium cathode tubes, acetylene gas for the measurement of absorbance, and a standard calcium solution with a 100 mg/L concentration-curve calibration adjustment of calcium ions. In AASF, the liquid sample is mixed with combustible gases like acetylene gas. Due to combustion, most relevant atoms of an element curtail to free unexcited ground-state atoms. Element-specific light is absorbed at a characteristic wavelength, which supplies a beam of light from the lamp. The cathode of the lamp is made up of elements to be tested, which pass across the flame. The amount of depletion in the light intensity is detected by a device called a photon multiplier device, which is due to the absorption of an analyte and is directly associated with the amount of element present in the sample to be tested. For the quantification of calcium ions by AASF, an individual value was established, and for each group, a mean value was used.

The data was evaluated using IBM SPSS Statistics for Windows, Version 22.0 (Released 2013; IBM Corp., Armonk, New York, United States). Means and standard deviations (SD) were determined. The normality of the data was assessed using the Kolmogorov-Smirnov test, and appropriate statistical tests were employed. A one-way analysis of variance (ANOVA) was used to correlate the mean calcium concentration among the groups. The Tukey post hoc test was applied to evaluate the variation in the mean calcium concentration between each group. A p-value <0.05 was taken into consideration as statistically significant at 95% CI.

## Results

The mean calcium concentration compared between the EDTA + laser group, EDTA alone group, chitosan + laser group, chitosan alone group, and control group showed values of 130.18, 121.13, 125.50, 118.64, and 0 mg/L, respectively. There was a statistically significant difference between the groups for the mean calcium concentration (p-value <0.001) (Table 1).

**Table 1:** Intergroup comparison of the mean concentration of calcium ions SD: standard deviation

Groups	Mean ± SD	p-value
1	130.18 ± 10.3	<0.001
2	121.13 ± 3.9	
3	125.50 ± 3.0	
4	118.64 ± 2.9	
5	0.00 ± 0.0	

The Tukey post hoc test was done to evaluate the multiple comparisons of the mean calcium concentration, which reported that there was statistically no significant difference between the EDTA + laser group and the chitosan + laser group (4.68 ± 2.0) (p-value = 0.21), whereas statistically significant difference was noted between the EDTA + laser group and EDTA alone group (9.05 ± 2.1) (p-value = 0.001), the EDTA + laser group and chitosan alone group (11.54 ± 2.2) (p-value <0.001), and the EDTA + laser group and control group (130.18 ± 2.1) (p-value <0.001) (Table 2).

**Table 2:** Multiple comparison of the mean calcium concentration between the groups SE: standard error

Groups		Mean difference ± SE	p-value
1	2	9.05 ± 2.1	0.001
	3	4.68 ± 2.0	0.21
	4	11.54 ± 2.2	<0.001
	5	130.18 ± 2.1	<0.001
2	3	-4.37 ± 2.1	0.273
	4	2.48 ± 2.2	0.783
	5	121.13 ± 2.0	<0.001
3	4	6.85 ± 2.0	0.02
	5	125.50 ± 2.1	<0.001
4	5	118.64 ± 1.9	<0.001

When compared with the control group, the mean calcium concentration was significantly higher among the EDTA alone group (121.13 ± 2.0) (p-value <0.001). Statistically, no significant difference was observed in the mean calcium ion concentration between the EDTA alone group and the chitosan + laser group (-4.37 ± 2.1) (p-value = 0.27) as well as between the EDTA alone group and chitosan alone group (2.48 ± 2.2) (p-value = 0.78). It was observed that there was a statistically significant difference in the mean calcium concentration between the chitosan + laser group and chitosan alone group (6.85 ± 2.0) (p-value = 0.02) as well as between the chitosan + laser group and control group (125.50 ± 2.1) (p-value <0.001). The mean calcium concentration between the chitosan alone group and the control group was significantly different between the groups (118.64 ± 1.9) (p-value <0.001).

## Discussion

The results of this AASF study showed that, except for group 5, all chelating agents were able to remove calcium ions from the root dentin. The values of 0.2% CNPs + Er:YAG laser and 15% EDTA + Er:YAG laser are greater than those without laser activation. This result is in line with the experiment done by Farag et al., who stated in their study that laser activation effectively removed the SL and debris from canal walls when employed along with 17% EDTA or 0.2% CNP chelating solutions, with the best response observed with 17% EDTA and laser [13]. The outcome of the present investigation showed that the Er:YAG laser enhanced the cleaning potential of chelating agents in the prepared root canals. It was also noted that there was a significant difference between the control group and the other groups that were activated with the laser.

Deleu et al. showed the high proficiency of Er:YAG laser-activated irrigation with a plain fiber tip in eliminating dentin debris from replicated root canal irregularities when Er:YAG laser-activated irrigation with a photon-induced photoacoustic streaming (PIPS) tip was compared to diode laser, conventional irrigation, and manual dynamic irrigation [14]. In our study, a similar protocol was followed where irrigants were activated using a plain fiber tip. In the present study, the control group did not elute any calcium, as only deionized water (without laser activation) was used as the final irrigant in this group. The statement is consistent with the research by Deleu et al. [14]. In the present study, the analysis showed a substantial difference in calcium ion elimination between group 5 (the control group) and other groups that were laser-activated. Identical results were noted in an scanning electron microscopy (SEM) analysis by Takeda et al., where the Er:YAG laser is efficient in cleaning the instrumented root canal compared to the control group [15].

In the current study, a laser tip was placed in the pulp chamber to activate the irrigants. This was done in line with previous research by Nagahashi et al., which showed that coronally placed laser tips effectively removed biofilm and showed excellent cleaning results, showing that irrigants activated with the laser have the ability to involve the action of the laser tip at a distance from the root canal for SL removal [16]. Added benefits of positioning the laser tip in the pulp chamber include agitation of the solution in the entire root canal system. This statement is in agreement with Kihara et al. Multiple canals can be treated simultaneously without damaging the periodontium or compromising the efficacy of the irrigants. It can also be used in narrow canals [9]. Therefore, in our study, we advocated this method of placing the laser tip in the pulp chamber. Though thermal changes have not been evaluated in our study, it has been observed in previous studies that placing the laser tip within the root canal has many drawbacks, such as the formation of ledges, charring of the root surface, fractures, and apical displacement of the irrigant [11]. In contrast to our study, results by Ashraf et al. demonstrated that EDTA removed SL better than the Er:YAG laser. A similar outcome was seen in the study by Ramalho et al. This deviating result could be due to the fact that the laser tip was placed at the working length and had not touched all the surfaces of the canal walls; as a result, SL was absent in the areas that had contact with the laser tip [10]. Laser-activated irrigation works on the principle of cavitation, where the cavitation bubbles expand and implode. The key role in SL removal in the root canal is the generation of shock, which diffuses the irrigants deeper into the tubules of the root dentin [11].

In this study, an Er:YAG laser was used to activate the irrigants. This is similar to an earlier study by Wigdor et al., in which they found that the Er:YAG laser caused less thermal damage than the neodymium-doped yttrium aluminum garnet (Nd:YAG) and carbon dioxide lasers [17]. The SL is generated irrespective of the type of instrument and methodology used [7]. Its presence in root canal walls will prevent the invasion of irrigants, therapeutic medications, and sealers from accessing the dentinal tubules. Its removal is essential as it harbors microorganisms and may directly lead to the failure of endodontic therapy [13]. Hence, in the current study, the removal of the SL was advocated by using the chelating agents activated with the Er:YAG laser. In the current study, 0.2% CNPs had a similar chelating effect as 15% EDTA-activated laser. Application time of three to five minutes is the most accepted duration to cause demineralizing efficacy of 0.2% CNPs on the root dentin [18]. Therefore, 0.2% CNP irrigant was employed for three minutes in our study. In an analysis done by Ratih et al., 0.2% CNPs used during the final irrigation had the same effect on the removal of SL when compared to 17% EDTA. These results are in accordance with our study, as observed by the results in groups 2 and 4. As shown in a previous study [19], 0.2% CNP is better than 17% EDTA because it raises the microhardness and makes the surface of the canal less rough.

CNPs and complexes of the metal ion are the consequences of ion exchange, chelation, and adsorption. The required ions, chitosan's chemical structure, and the solution's pH determine the type of interaction [20]. Lottanti et al. have highlighted the pitfalls of SEM analysis to evaluate the existence of a SL in their study. According to those authors, there is no clear distinction between the SL and sclerotic dentin when examined under SEM [21]. Earlier research with flame AASF also revealed that the use of chelating agents was able to remove calcium ions from the root canals [22]. Hence, calcium ion quantification by the AASF study has been adopted in this study to assess the removal of the SL by the chelating agents activated with the Er:YAG laser. In our study, AASF analysis showed that, except for the control group, all solutions tested eluted calcium ions from the root dentin. After irrigation, the calcium ions in these solutions are not solely from the decalcification of inorganic parts in the SL. There will be exposure to collagen and reduced microhardness due to the action of these chelating and demineralizing agents on the hydroxyapatite calcium matrix of the root. Therefore, it was depicted that the irrigating solution with a greater calcium ion concentration was considered to have the potential for demineralization and higher cleaning efficacy [20].

For efficient debris removal and deeper penetration of irrigant, it must contact dentin walls, making more surface area available for its action [7]. In our study, CNPs were used because of their greater absorption and intense penetration into the dentinal tubule. As a result, there was improved SL removal. This statement is consistent with the investigation by Ratih et al. The hydrophilic nature of chitosan causes it to be rapidly absorbed into root canal dentin with deep penetration into dentinal tubules [23]. EDTA has greater potential to demineralize the SL, especially the inorganic content, due to which it is considered a strong chelating agent. This demineralization effect has the drawback of peritubular and intertubular dentin erosion, which results in enlarged dentinal tubules, weakening, and alteration of the dentinal surface, which is due to the alteration in the calcium/phosphate ratio in dental hard tissue. This alteration leads to decreased microhardness and increased surface roughness of the root dentin [19].

In contrast to EDTA, in a study done by Ratih et al., 0.2% CNPs showed lesser structural changes in dentin, which proves CNPs to be a weak chelating agent with less demineralization capacity but effective SL removal. Moreover, chitosan has the added advantage of inducing the remineralization of demineralized dentin due to its covalent bonding to dentinal collagen, with increased microhardness and decreased surface roughness [19]. In the current study, chelating agents were chosen as irrigants due to their benefits in routine clinical practice, which facilitate the biomechanical preparation of tapered or obstructed canals, thereby improving the binding of the endodontic sealer to the root canal by providing the surface roughness of the dentin required for sealer retention [23]. The proficiency of a chelating agent depends on several factors, like time of application, pH, concentration, and amount of the irrigant. Thus, in this study, final irrigation was used for three minutes with a volume of 5 mL, which is in accordance with previous studies [19,24].

In the present study, the decalcifying features of two chelating agents were examined, which were activated with an Er:YAG laser. When chelating agents interact with dentin, there is the release of calcium ions from the dentin, which increases dentin permeability and hence plays a vital role in the removal of the SL [24]. The productiveness of 0.2% CNPs and 15% EDTA, both activated with laser, was assessed depending on the quantity of calcium ions released using AASF with flame, which permits quantifying the calcium ion concentration present in the solution after irrigation, in accordance with previous authors [7,20]. The chelating agent alone (EDTA) or the laser action alone for SL removal was ineffective in earlier studies [25,26]. In this study, though EDTA alone and chitosan alone have the same efficacy in removing calcium ions from the root dentin, it must be noted that the mean calcium ion concentration of laser-activated EDTA and CNPs is higher. This proves that laser activation of irrigants has superior action over chelating agents when used alone.

In the present study, the mean calcium concentration of CNPs with the laser activation group is higher than that of the EDTA alone group. Although there was statistically no significant difference between these two groups, which produced similar effects for calcium ion removal, less concentrated solutions are preferable [27]. Moreover, chitosan is abundantly found in nature and is also economical [20]. The scarcity of research addressing the use of laser-activated 0.2% CNPs and 15% EDTA activated with Er:YAG laser hampers the comparative observation of these findings to previous studies. Taking into account the gratifying outcome established in this study with 0.2% CNP solution in combination with laser activation and the merits of this biological polymer over EDTA [19], further in vivo studies are essential to probe the use of this chelating agent in endodontics as a final irrigant.

## Conclusions

In this study, the results revealed that the combination of 0.2% CNPs with laser activation demonstrated comparable calcium ion release efficiency to that achieved by using a higher concentration of 15% EDTA. This finding highlights the potential of 0.2% CNPs as a promising alternative solution to traditional EDTA for the removal of calcium ions in root canals. Moreover, the superior performance of 0.2% CNPs when activated with the Er:YAG laser suggests a more effective and efficient approach to calcium ion removal during root canal treatment. This innovative combination not only offers a viable substitute for EDTA but also presents a promising avenue for enhancing the overall efficacy of root canal procedures, potentially improving patient outcomes and treatment success.
